# Electronic health record risk-stratification tool reduces venous thromboembolism events in surgical patients^☆^

**DOI:** 10.1016/j.sopen.2022.04.003

**Published:** 2022-04-26

**Authors:** Radhika Rastogi, Courtney M. Lattimore, J. Hunter Mehaffey, Florence E. Turrentine, Hillary S. Maitland, Victor M. Zaydfudim

**Affiliations:** a Department of Surgery, University of Virginia, Charlottesville, VA 22908; b Surgical Outcomes Research Center, University of Virginia, Charlottesville, VA 22908; c Department of Medicine, Hematology/Oncology, University of Virginia, Charlottesville, VA 22908

**Keywords:** EHR, electronic health record, SCD, sequential compression devices, VTE, venous thromboembolism, Venous thromboembolism reduction, Risk stratification tool, EHR clinical decision support, Electronic dashboard

## Abstract

**Background:**

Venous thromboembolism is a preventable cause of morbidity and mortality after surgery. To ensure that patients receive appropriate venous thromboembolism chemoprophylaxis, a nonmandatory risk-stratification tool based on patient clinical condition was implemented through the electronic health record to stratify patient risk and recommend chemoprophylaxis. We hypothesized that implementing this tool would reduce postoperative venous thromboembolism events in general surgery as well as across all surgical services.

**Methods:**

All adult patients undergoing inpatient surgical operations (January 2012–December 2019) at a single quaternary care center and Level 1 trauma center were abstracted from institutional electronic health record database and stratified into patients admitted before and after venous thromboembolism risk-stratification tool implementation. Bivariable analyses compared venous thromboembolism chemoprophylaxis prescription and venous thromboembolism events with implementation and screening among all surgical patients as well as in general surgery patient subset.

**Results:**

A total of 64,377 adults underwent operations: 27,819 preimplementation and 36,558 postimplementation. A significant reduction in venous thromboembolism events occurred from pre- to post-tool implementation for all cases (0.77% vs 0.47%, P < .001). General surgery patients (*n* = 15,723) had a significant increase in chemoprophylaxis prescription (81.9% vs 86.0%, P < .001) and a significant reduction in venous thromboembolism events (1.41% vs 0.59%, P < .001). After tool implementation, use of extended postdischarge chemoprophylaxis was greater among general surgery patient subset than the entire patient cohort (46.7% vs 29.6%, P < .001).

**Conclusion:**

The integration of a nonmandatory electronic health record risk-stratification tool was associated with a significant reduction in venous thromboembolism events. Extended chemoprophylaxis was prescribed in nearly half of general surgery patients at very high risk for postdischarge events.

## Highlights

• Implementing an electronic VTE risk-stratification tool reduced surgical VTE events.
• Even as a nonmandatory tool, risk stratification led to overall fewer VTE events.
• Postoperative VTE events were reduced by 39% after the tool was integrated in EHR.
• With the tool, general surgery had 58% less VTE events and improved prophylaxis use.

## INTRODUCTION

Venous thromboembolism (VTE) affects up to 20%–30% of surgical patients with significant morbidity and mortality [1]. A third of deaths related to VTE occur in the postoperative period [2]. The Agency for Healthcare Research and Quality, American College of Chest Physicians, American Heart Association, and The Joint Commission have all identified VTE as a primary quality measure to address through appropriate mechanical and pharmacologic prophylaxis as it remains among the most preventable complications [[2], [3], [4], [5]]. With the use of appropriate chemoprophylaxis, VTE in the postoperative period can be reduced by up to 75% [6,7].

To determine appropriate VTE prophylaxis recommendations in surgical patients and to minimize VTE events, the American College of Chest Physicians has established the evidence-based CHEST guidelines to identify at-risk patients based on risk stratification and to specify corresponding appropriate prophylaxis based on stratification category [2,8]. Risk stratification is determined with risk assessment models, such as the Caprini or Rogers scores, which assess patient VTE risk factors and procedural variables to classify patients as low, moderate, high, or very high risk for a VTE event [2,[9], [10], [11], [12]]. Despite establishment of guidelines and encouragement from numerous surgical societies [13,14], clinicians do not consistently identify at-risk patients and frequently do not select appropriate chemoprophylaxis. Inappropriate chemoprophylaxis including erroneous dose prescription or failure to order any chemoprophylaxis occurs for up to 40%–50% of surgical patients [6,15,16]. There has been increasing use of risk assessment models to aid in identification and stratification of at-risk patients [17]. Additionally, the use of electronic clinical decision-making tools has evolved as a promising modality in standardizing and improving appropriate prophylaxis and reduction of VTE events [15,[18], [19], [20], [21], [22], [23], [24], [25]].

To date, published data have evaluated mandatory electronic stratification tools in either limited service-specific populations or in large populations of combined medical and surgical patients. Identification of VTE prevention in a surgical patient population as a critical patient-specific quality measure at our institution resulted in the creation of an electronic VTE risk-stratification tool which was implemented in a rolling fashion between 2014 and 2017 across all surgical services. This study aimed to analyze the impact of a nonmandatory VTE risk-stratification tool on VTE events across all surgical and surgical subspecialty services over 5 years, including the 3-year rolling implementation period. We hypothesize that tool implementation would increase VTE chemoprophylaxis prescription and result in fewer VTE events across all surgical specialties and specifically general surgery. We further hypothesize that increased tool utilization, once available, would reduce overall VTE events.

## MATERIALS AND METHODS

### Clinical Setting and Patient Population

This retrospective cohort study was performed at the University of Virginia Medical Center, an academic quaternary care and Level 1 trauma center. The study population included all adult patients (≥ 18 years of age) who underwent an inpatient operation between January 1, 2012, and December 31, 2019, in the following surgical departments/divisions: Neurosurgery, Thoracic Cardiovascular Surgery, Obstetrics and Gynecology, Orthopedic surgery, Otolaryngology, Plastic Surgery, General Surgery, and Urology. Further analysis was done with general surgery patients due to high patient volumes and high VTE event risk.

### Study Design

The objective of this study was to compare VTE outcomes among surgical patients before and after the initiation of a nonmandatory VTE risk-stratification tool. The primary outcome measure was *inpatient VTE events*, defined as any pulmonary embolism or deep venous thrombosis diagnosed during the inpatient admission period for each patient by either symptomatic workup or incidental imaging. Secondary outcomes were defined as prescription of VTE chemoprophylaxis, choice of VTE chemoprophylaxis medication, prophylaxis prescribed on discharge in the very high risk patient subgroup, and compliance with EHR tool utilization.

All patients undergoing an operative procedure in the defined surgical specialties were abstracted from the EHR (Epic) from 2012 through 2019. Patient variables included age, race, sex, operating service, length of stay, VTE chemoprophylaxis prescribed, and inpatient VTE events. Patients were categorized into pre- or postimplementation eras based on the date the tool was implemented in the specific surgical service admission order set. Analysis comparing VTE events, VTE chemoprophylaxis prescription, and prophylaxis ordered at discharge for very high risk patients in the pre- and postimplementation eras was performed separately for all surgical services and general surgery patients. Once the tool was implemented and available within the EHR, compliance with tool utilization and its impact on VTE events were also analyzed.

### Electronic Risk-Stratification Tool

**Fig 1:**
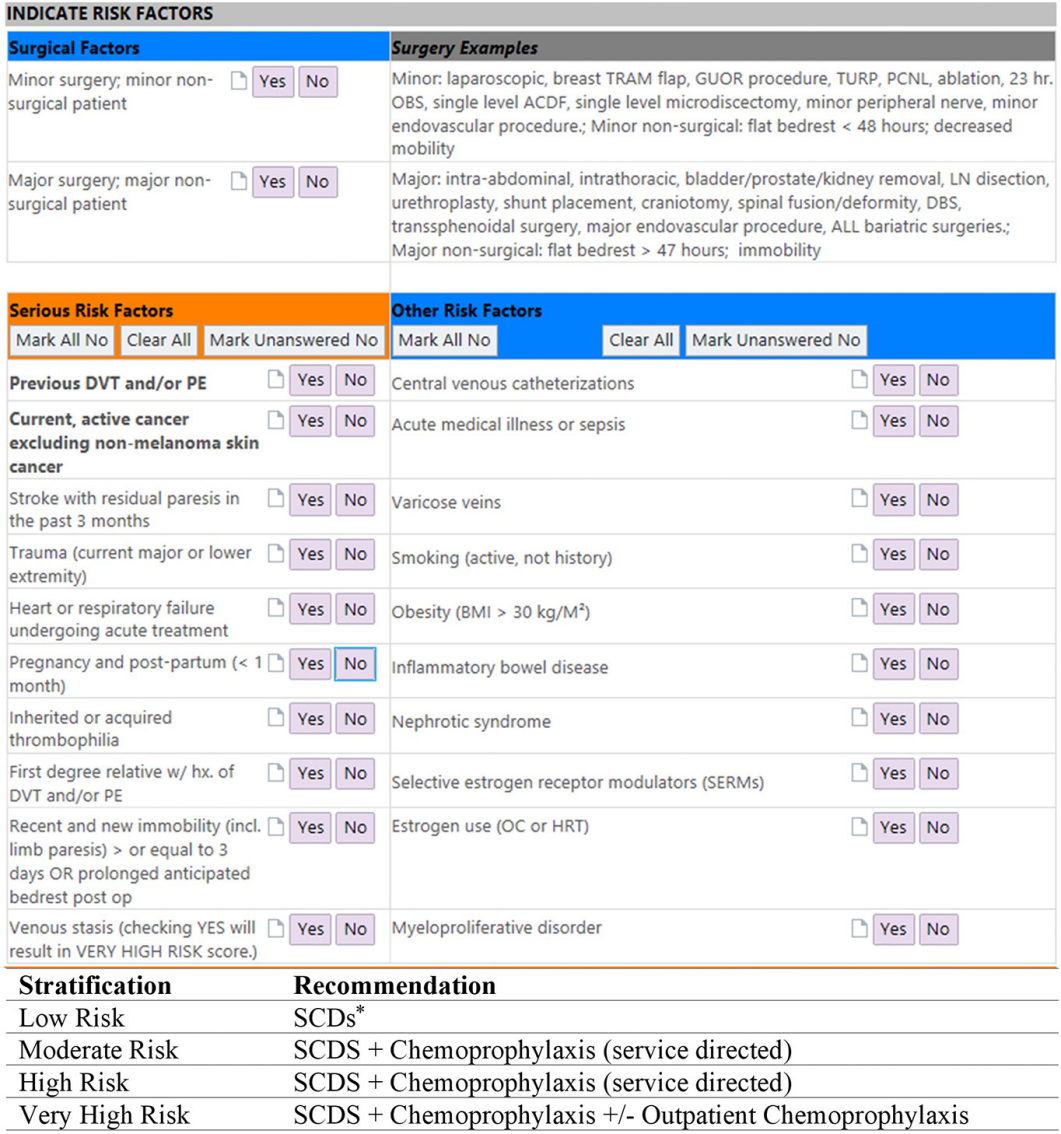
Venous thromboembolism risk-stratification tool. Interface of venous thromboembolism risk-stratification tool, using surgical factors, clinical risk factors from patient history, and current clinical condition to stratify patients into low-, moderate-, high-, and very high risk categories.

The VTE risk-stratification tool for surgical patients was developed in 2014 and implemented in a rolling fashion (through 2017) within each service line's admission order sets. The tool adjusted for patient risk factors, current clinical condition, and surgical factors after quick manual entry of "yes" or "no" for the various clinical factors by clinicians. The EHR tool was based upon the modified Johns Hopkins Hospital mandatory decision support tool, a validated tool that was derived and condensed from the Caprini score, which assessed 13 factors: previous VTE, cancer, thrombophilia, prolonged procedure > 2 hours, New York Heart Association Class III/IV heart failure, respiratory failure requiring mechanical ventilation, acute stroke with paresis < 3 months, pregnancy/postpartum state, acute infection/sepsis, bed rest, central venous catheter presence, estrogens/estrogen receptor, and inflammatory bowel disease [23,26]. Additional evidence-based variables were added based on consensus from our institutional VTE Reduction Taskforce, including myeloproliferative disorder, nephrotic syndrome, body mass index > 30, active smoking, major trauma, venous stasis, and a first-degree relative with history of VTE **(**Fig 1**)** [10,12]. Operations were stratified as major or minor, and subsequent patient factors were categorized as major (ie, trauma, active cancer, prior VTE) and minor (ie, smoking, body mass index, inflammatory bowel disease) to categorize patients into risk groups.

The tool was completed upon admission after surgery, requiring approximately 1–2 minutes, and based on the tool's computerized algorithm, patients were stratified into 4 categories: low risk, moderate risk, high risk, and very high risk. After reviewing current clinical practice guidelines for adult VTE prophylaxis for their respective specialties, surgeon consensus from each individual surgical service line determined if chemoprophylaxis was indicated, which drug to prescribe, and appropriate weight-based dosing for each risk category [2,8,27]. These recommendations were then integrated within the admission order sets for service-specific standardization. For all categories, mechanical prophylaxis with sequential compression devices (SCDs) or compression stockings was recommended. For moderate- and high-risk patients, VTE chemoprophylaxis was recommended in addition to mechanical prophylaxis, and for those at very high risk, additional outpatient chemoprophylaxis for 4 weeks after discharge was strongly encouraged [28]. Once a patient was appropriately stratified, the admission EHR order set would only provide prophylaxis options of the stratified category with the drug options and dosing determined by that service. Enoxaparin was recommended unless patients had renal dysfunction, and heparin or apixaban options were available, particularly if creatinine clearance was < 30 mL/min. Thus, the risk-stratification EHR tool provided clinicians concrete options to guide drug and dosing options for all patients. The tool was an integrated step in admission and transfer order sets and provided a recommendation at that time point.

However, an EHR mandatory hard-stop to ensure completion of the VTE screening tool within surgical order sets was not an option at the time of the tool build. Instead, clinician education and integration in workflow were utilized to encourage screening completion. Initial education was provided during the pilot period of the tool implementation. An open house with EHR experts was held to guide clinicians on how to stratify patients and use the tool. Education sessions were held for residents and surgeons, resident "champions" were trained to guide other residents in the process, and real-time assistance was provided during rounds. Compliance was initially tracked with clinicians notified by email when issues arose, and further step-by-step instructions on tool usage was provided. After successful rollout of the tool and education across services, subsequent education was from fellow residents during initial onboarding and with periodic email instructions on screening completion.

### Statistical Analyses

Categorical data was presented as *n* (%) and compared using *χ^2^* test in each specified group in pre- and postimplementation eras as well as in the postimplementation era, comparing patients who were or were not screened. Continuous data were presented as mean ± standard deviation for normally distributed data and median with interquartlie range for non-normal data distributions. Comparisons were performed with Student *t* test or Wilcoxon rank-sum test as appropriate. All statistical analyses were performed using SAS Version 9.4 (SAS Institute, Cary, NC). This protocol was approved by the Institutional Review Board for Health Sciences Research (University of Virginia #20268).

## RESULTS

### Inpatient VTE Events

A total of 64,377 adults had an inpatient operation during the study period, 43% preimplementation and 57% postimplementation. Proportions of male patients in pre- and postimplementation eras were 50.7% and 53.5%, respectively. In both pre- and postimplementation eras, the patient population was predominantly white (85.2% and 83.3%, respectively) with a mean age of 56 years (SD 17.5) and 57 years (SD 17.5), respectively (Table 1**)**. Of these, there were 15,723 general surgery patients, with nearly 60% in the postimplementation era.

**Table 1:** Demographic and clinical covariate (patient demographics and outcomes for all surgical cases and general surgery)

*Variable*	*Preimplementation*	*Postimplementation*	P *value*
Population			
All surgical cases	27,819 (43.2%)	36,558 (56.8%)	
General surgery	6,610 (42.0%)	9,113 (58.0%)	
Mean age	56 (SD 17.5)	57 (SD 17.5)	*P* < .0001
Sex			*P* < .0001
Male	14,098 (50.7%)	19,571 (53.5%)	
Female	13,721 (49.3%)	16,987 (46.5%)	
Race			*P* < .0001
White	23,698 (85.2%)	30,439 (83.3%)	
African American	3,309 (11.9%)	4,664 (12.8%)	
Asian	155 (0.6%)	281 (0.8%)	
Other	657 (2.4%)	1,174 (3.2%)	
VTE⁎ medication administered			*P* < .0001
None	6,097 (21.9%)	9,165 (25.1%)	
Enoxaparin	7,332 (26.4%)	11,003 (30.1%)	
Heparin	12,248 (44.0%)	14,386 (39.4%)	
Warfarin	2,049 (7.4%)	1,347 (3.4%)	
Apixaban	27 (0.10%)	410 (1.1%)	
Rivaroxaban	38 (0.14%)	221 (0.60%)	
Other	28 (0.10%)	26 (0.07%)	
VTE events	214 (0.77%)	171 (0.47%)	*P* < .0001
LOS⁎ (d)	5.4 (SD 7.1)	5.2 (SD 6.6)	*P* < .0001

⁎ LOS, length of stay.

**Fig 2:**
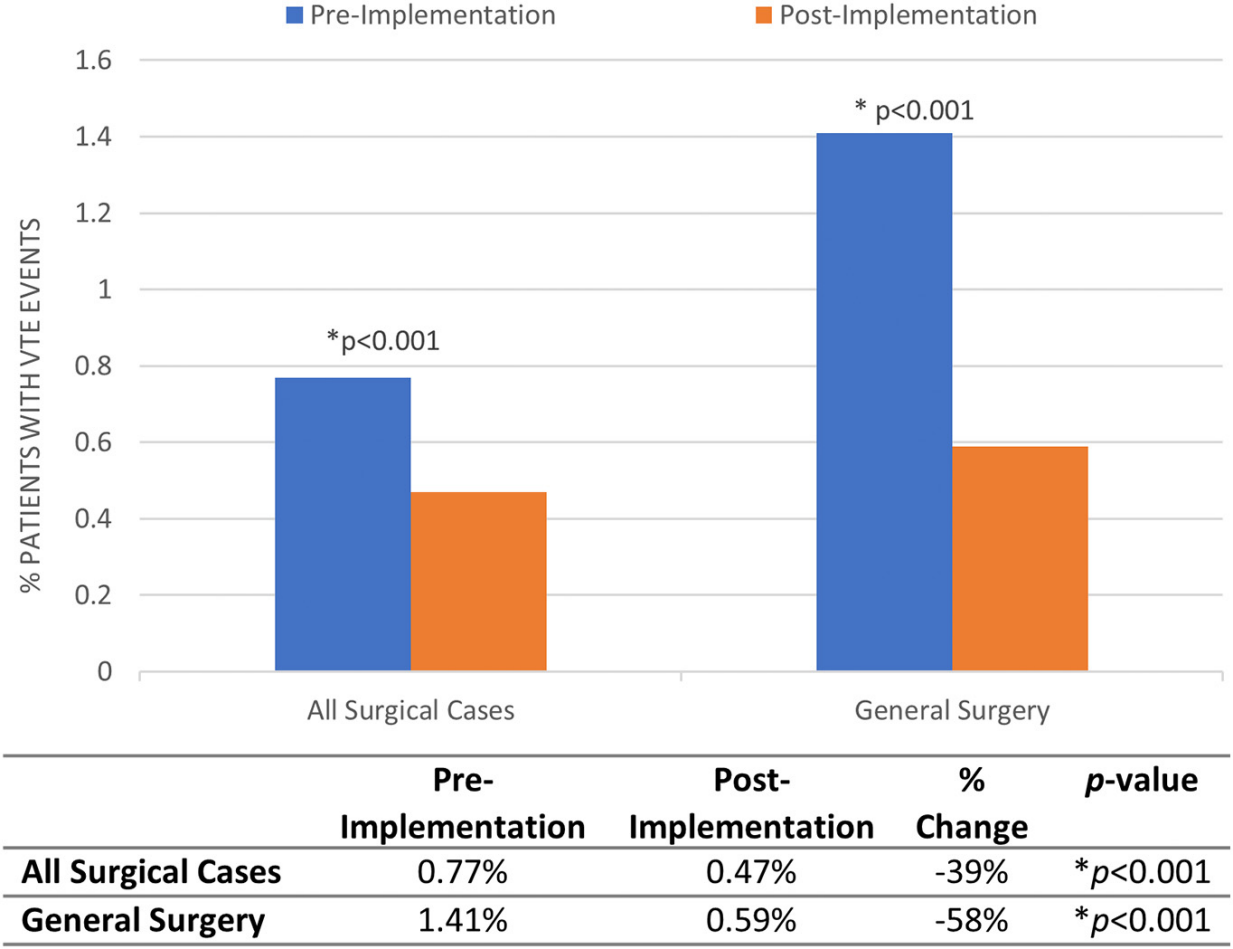
Venous thromboembolism events across pre- and postimplementation eras. Comparison of proportion of patients with venous thromboembolism events between pre- and postimplementation eras for all surgical cases and general surgery. *Significance of *P* < .05.

VTE event rate was 0.77% in the preimplementation era (214 patients) (Table 1**)**. Implementation of the risk-stratification tool was associated with a 39% reduction of VTE events to 0.47% event rate (171 patients, *P* < .001) across all surgical services **(**Fig 2**)**. General surgery patients had a similarly significant 58% reduction in VTE events (1.41% vs 0.59%, *P* < .001).

### Compliance with EHR Tool Postimplementation

**Fig 3:**
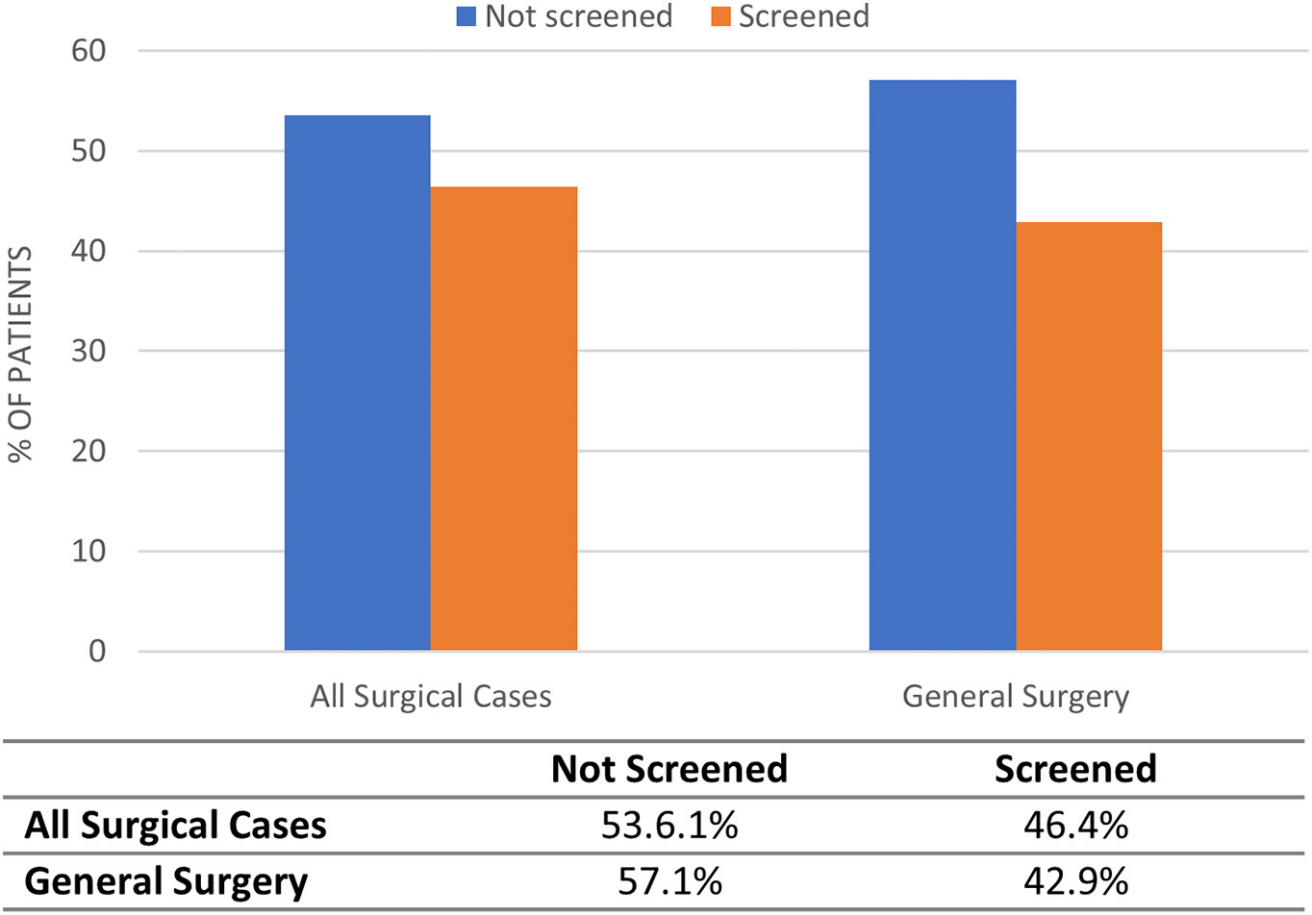
Proportion of tool utilization in postimplementation era. Comparison of the proportion of patients in the postimplementation era who did or did not have the venous thromboembolism risk-stratification tool used and screening completed in all surgical cases and general surgery. *Significance of *P* < .05.

**Fig 4:**
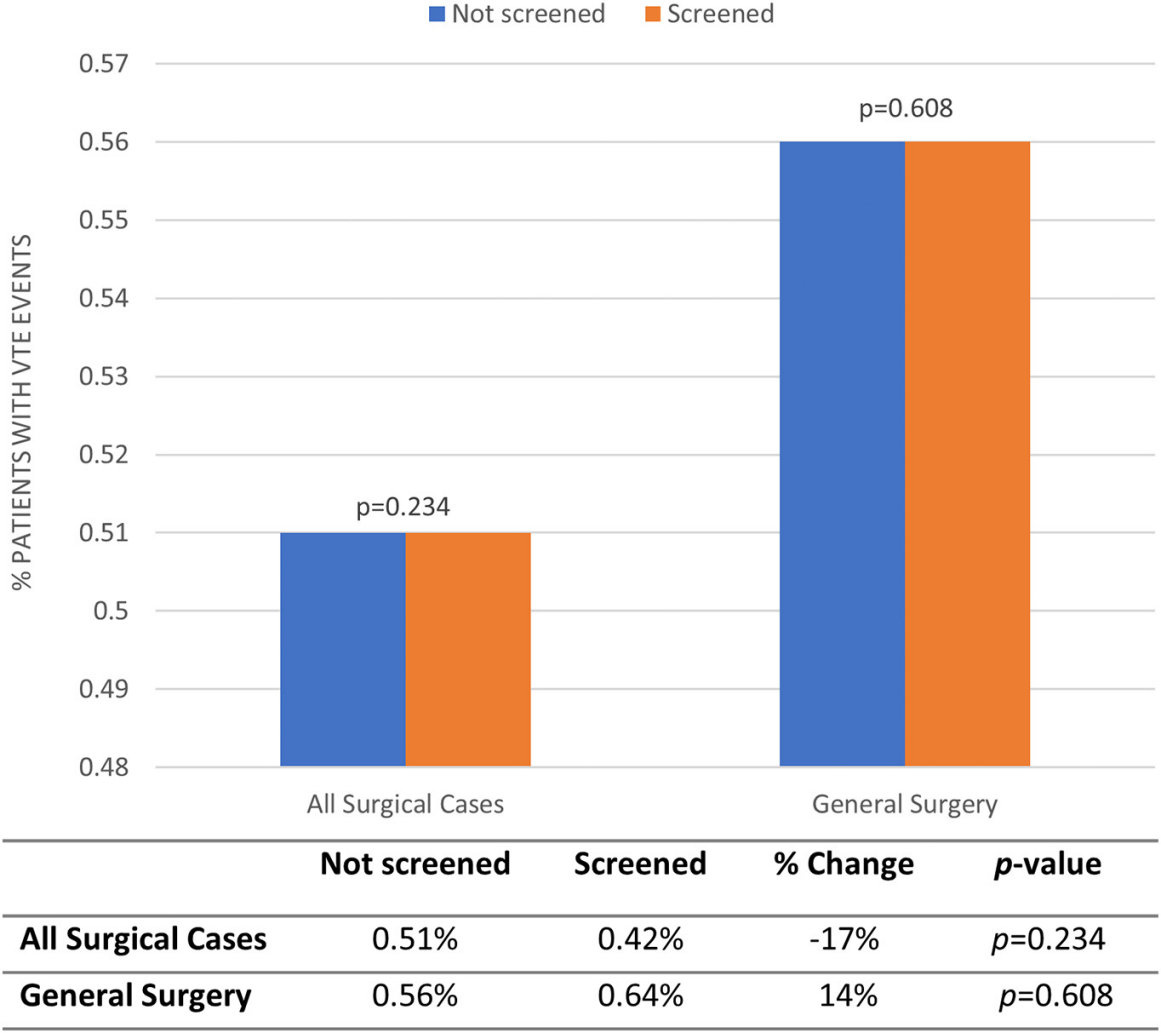
Postimplementation era venous thromboembolism events with tool utilization. Comparison of proportion of patients with venous thromboembolism events in the postimplementation era who did or did not have the risk stratification tool used and screening completed for all surgical cases and general surgery. *Significance of *P* < .05.

Overall compliance with EHR tool utilization was moderate in the postimplementation era, with 46.4% of all surgical patients and 42.9% of general surgery patients completing EHR screening **(**Fig 3**)**. Postimplementation, there was no difference in VTE events between patients who were not screened versus screened with the EHR tool (0.51% vs 0.42%, respectively, *P* = .234) (Fig 4). Similarly, there was no significant difference in VTE events in patients by EHR screening in general surgery (0.56% vs 0.64%, *P* = .61).

### VTE Chemoprophylaxis

**Fig 5:**
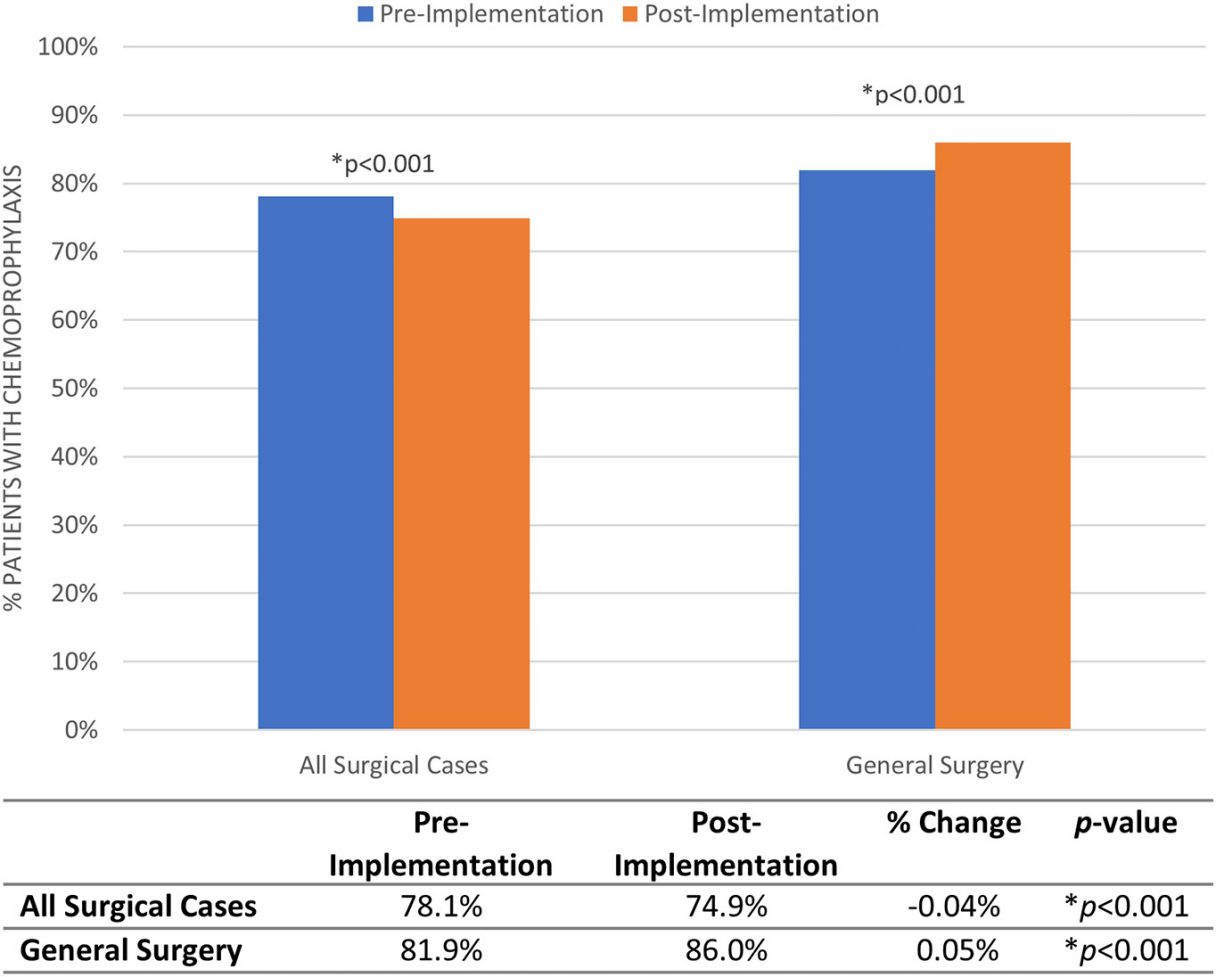
Venous thromboembolism chemoprophylaxis prescription. Comparison of the proportion of patients receiving venous thromboembolism chemoprophylaxis in the pre- versus postimplementation eras for all surgical cases and general surgery. *Significance of *P* < .05.

The proportion of VTE chemoprophylaxis prescription increased significantly from the pre- to postimplementation era for general surgery (81.5% *P* =  86.0%, *P* < .001) (Fig 5). Heparin was the most common prophylaxis medication administered for all surgical cases (44.0% vs 39.4%, *P* < .001) (Table 2). In general surgery patients, enoxaparin was used with the greatest frequency (52.5% vs 61.4%, *P* < .001) in pre- and postimplementation eras. There were fewer VTE events in patients receiving enoxaparin (0.60% vs 0.93% compared to heparin, *P* < .001).

**Table 2:** Venous thromboembolism medication prescribed (distribution of venous thromboembolism chemoprophylaxis medications prescribed in the preimplementation [Pre] versus postimplementation [Post] eras for all surgical cases and general surgery)

	*Era*	*None*	*Enoxaparin*	*Heparin*	*Warfarin*	*Apixaban*	*Rivaroxaban*	*Other*	P *value*
All surgical cases	Pre	6,097 (21.9%)	7,332 (26.4%)	12,248 (44.0%)	2,049 (7.4%)	27 (0.10%)	38 (0.14%)	28 (0.10%)	*P* < .0001
	Post	9,165 (25.1%)	11,003 (30.1%)	14,386 (39.4%)	1,347 (3.4%)	410 (1.1%)	221 (0.60%)	26 (0.07%)	
General surgery	Pre	1,194 (18.1%)	3,468 (52.5%)	1,903 (28.8%)	29 (0.44%)	5 (0.08%)	6 (0.09%)	5 (0.08%)	*P* < .0001
	Post	1,279 (14.0%)	5,592 (61.4%)	2,176 (23.9%)	25 (0.27%)	31 (0.34%)	9 (0.10%)	1 (0.01%)	
VTE events		9 (0.06%)	110 (0.60%)	249 (0.93%)	14 (0.41%)	3 (0.69%)	0 (0.0%)	0 (0.0%)	*P* < .0001

Among the 5,051 patients who met the very high risk criteria across all surgical cases, 29.6% were prescribed additional chemoprophylaxis upon discharge. Outpatient prophylaxis was prescribed at a greater rate of 46.7% among general surgery patients identified as very high risk compared to all surgical cases (*P* < .001).

## DISCUSSION

The introduction of a VTE risk-stratification tool within the EHR resulted in a significant decrease in VTE events across all surgical patients and among the general surgery patient subgroup in the 5-year period after implementation. This study demonstrates applicable benefit of the VTE stratification tool across all surgical and surgical subspecialty departments by standardizing individual patient risk assessment and VTE prophylaxis prescription on admission.

Interestingly, tool utilization in the postimplementation era did not lead to a significant difference in VTE events between those who were and were not screened for all surgical cases as well as for general surgery patients. This was particularly notable as use of the risk-stratification tool itself was not mandatory and approximately half of the patients did not undergo screening after tool implementation. Unlike studies with a mandatory VTE stratification tool [19,22,23], our risk-stratification tool could not be mandatory because of limitations in the EHR build during the implementation period. However, our data demonstrate substantial benefit even in the nonmandatory tool utilization setting. Possible reasons for moderate compliance with tool utilization include (1) decreased clinician education after the tool had been well established in order sets and (2) anticipation of patient risk level without tool usage and selection of prophylaxis accordingly, which may contribute to noncompliance with tool utilization without impact on VTE events. Nevertheless, standardization of tool utilization should optimize VTE prophylaxis for surgical patients. Further targeted efforts toward improving tool compliance, including annual clinician education with new residents, screening integration into admission workflow, "opt-out" chemoprophylaxis prescription, automation of risk stratification by EHR data, and alternate methods to mandating the screening, are currently in development and are likely to increase compliance [20,23,26,[29], [30], [31]]. Moreover, addition of automatic EHR data collection to prefill parts of the tool as well as EHR prompts to update screening with patient condition would further benefit this process.

The addition of decision support tools to initiate chemoprophylaxis has aided in increasing chemoprophylaxis and reducing VTE events [[18], [19], [20], [21], [22], [23], [24]]. Johns Hopkins Hospitals' implementation of paper and then computerized physician decision support tools increased chemoprophylaxis nearly 3-fold from 2005 to 2011 for surgical patients (26% vs 80.2%) [23,26]. Moreover, a meta-analysis of 11 studies regarding the efficacy of computerized clinical decision support systems for VTE chemoprophylaxis demonstrated a significant increase in appropriate chemoprophylaxis ordered (odds ratio [OR] 2.35, 95% confidence interval, 1.78–3.10; *P* < .001) and significant decreases in VTE events (relative risk [RR] 0.78; 95% CI, 0.72–0.85; *P* < .001) [19]. Within our study, we were able to achieve similar significant reductions in VTE events for all surgical patients (0.77% vs 0.47%, *P* < .0001). Our study demonstrated persistent reduction in inpatient VTE events over a 5-year period in the largest single hospital cohort to date with a nonmandatory but well-integrated VTE tool.

Although VTE events decreased, results were more varied with respect to prescribing chemoprophylaxis. There was a decrease in prescription of VTE chemoprophylaxis among all surgical patients, with a significant increase in chemoprophylaxis among general surgery patients. The decrease in prescription with all cases may be in part due to specific subspecialty subset practices. For instance, this may be due to the recent increasing prevalence of aspirin as postoperative VTE chemoprophylaxis in specific orthopedic populations [8,32]; as use of aspirin was not reviewed within this study, orthopedic patients managed with aspirin appear as if they did not receive prophylaxis. Furthermore, these contrasting results with the same reduction in primary outcome may also reflect that the VTE risk-stratification tool was able to guide individualized, judicious prophylaxis prescription while also identifying patients who would benefit from not receiving chemoprophylaxis. As reported in Table 2, only 0.06% of patients who did not receive VTE chemoprophylaxis had VTE events, indicating that these patients were at a very low risk. A recent meta-analysis has demonstrated that chemoprophylaxis provides no definitive benefit for patients stratified to lower risk categories for VTE [11], suggesting that appropriate identification of lower risk patients may contribute to decreased chemoprophylaxis prescription with decreased VTE events.

Choice of chemoprophylaxis did not change with tool implementation, with heparin and enoxaparin as the primary drugs of choice. Heparin was most commonly prescribed across all services and enoxaparin for general surgery. Enoxaparin use had lower VTE events (0.60% vs 0.93% when compared to heparin, *P* < .0001). Prior studies, particularly in trauma populations, have demonstrated preference for enoxaparin as a more effective chemoprophylaxis [13,33,34].

One of the challenges in prescribing chemoprophylaxis is balancing the risk of VTE with the risk of clinically significant postoperative bleeding. Within general surgery, chemoprophylaxis is avoided in breast surgery and thyroid/parathyroid surgery because of the risk of hematoma or bleeding outweighing VTE reduction in this lower-risk population [35,36]. In addition, lower-risk patient populations often do not have their screening completed, contributing to tool noncompliance. Nevertheless, although some studies have suggested an increase in risk of bleeding and hematoma with use of chemoprophylaxis [1,2,11], the absolute risk remains very low, with discontinuation of prophylaxis in less than 2% of patients and very rare need for reoperation [1,37].

There are also well-established data for 4 weeks of extended postoperative VTE chemoprophylaxis in very high risk patients, although implementation of postdischarge prophylaxis remains controversial [2,28]. In this study, less than half of patients who were stratified to this category received recommended postdischarge chemoprophylaxis. One of the potential explanations could be attributed to individual clinician judgment weighing the risks of extended chemoprophylaxis to potential benefit. In addition, outpatient cost of novel oral anticoagulants or enoxaparin could be prohibitive, and use of enoxaparin injections is inconvenient [27,38].

Our study contains several limitations. The primary outcome measure was inpatient VTE events found based on symptomatic workup or incidental imaging, which was collected for all patients in the study. Longer-term VTE events were not granularly collected for the study population. The analysis also did not adjust for patient factors and comorbidities, although such factors were integrated within the scoring tool itself. Future analysis adjusting for patient comorbidities may be able to identify specific patient groups that would most benefit from tool utilization, chemoprophylaxis, and use of extended prophylaxis. Moreover, aspirin was not abstracted as a chemoprophylaxis agent, although it was used in select orthopedic surgery patient cohorts. We also could not systematically collect or analyze SCD use which was frequent in most postoperative inpatients and ordered at all levels of stratification. Given that the study period spans 8 years, changes in standard clinical practices, clinical and temporal changes, and other interventions beyond the implementation of the tool may also have impacted results. VTE reduction with tool implementation despite incomplete tool utilization may also suggest that the presence of the tool itself may result in practice changes and decreased VTE events even without tool completion, mirroring, in part, the efficacy of alerts in reducing VTE events [20].

In conclusion, the integration of an EHR VTE risk-stratification tool is associated with significant reduction in VTE events across surgical patients and remains an effective and promising intervention across all surgical and surgical subspecialty services. The tool effectively screened patients into appropriate risk categories and allowed for appropriate selection of VTE prophylaxis, providing a standardized method for decisions and dosing in the postoperative period. Even when the tool is not mandatory, it is able to effectively reduce overall VTE events. This tool, in combination with clinical decision making, aids in mitigating VTE events in the postoperative period. Ensuring full compliance with tool utilization and appropriately prescribing VTE chemoprophylaxis at discharge to high risk patients will likely further reduce VTE events.

## Author Contribution

Radhika Rastogi: Conceptualization, Methodology, Data interpretation, Visualization, Writing – original draft, Writing – review & editing.

Courtney M. Lattimore: Conceptualization, Data interpretation, Writing – original draft,

James Hunter Mehaffey: Conceptualization, Methodology, Data curation, Formal analysis, Data interpretation, Writing – review & editing.

Florence E. Turrentine: Conceptualization, Methodology, Data curation, Data interpretation, Writing – review & editing.

Hillary S. Maitland: Conceptualization, Data interpretation, Writing – review & editing.

Victor M. Zaydfudim: Funding acquisition, Supervision, Data interpretation, Writing – review & editing.

## Conflict of Interest

None of the authors have any direct or indirect financial conflicts of interest to disclose.

## Funding Source

This work was supported in part by the 10.13039/100000054National Cancer Institute(award 2L30 CA220861-02A2) to Victor M. Zaydfudim.

## Ethics Approval

This protocol was approved by the Institutional Review Board for Health Sciences Research (University of Virginia #20268).
